# Interactions of Functionalized Multi-Wall Carbon Nanotubes with Giant Phospholipid Vesicles as Model Cellular Membrane System

**DOI:** 10.1038/s41598-018-36531-9

**Published:** 2018-12-20

**Authors:** Verónica Pérez-Luna, Carlos Moreno-Aguilar, José Luis Arauz-Lara, Said Aranda-Espinoza, Mildred Quintana

**Affiliations:** 1 Instituto de Física, Universidad Autónoma de San Luis Potosí, Manuel Nava 6, Zona Universitaria, 78290 San Luis Potosí, SLP Mexico; 2 Centro de Investigación en Ciencias de la Salud y Biomedicina, Universidad Autónoma de San Luis Potosí, Av, Sierra Leona 550, 78210 San Luis Potosí, SLP Mexico

## Abstract

Carbon Nanotubes (CNTs) are considered alternative materials for the design of advanced drug and gene delivery vectors. However, the mechanism responsible for the cellular membrane intake of CNTs is not well understood. In the present study, we show how multi-walled carbon nanotubes (MWCNTs) owning different surface properties, interact with giant unilamellar vesicles (GUVs), a simple model system for cellular membranes. In particular, we want to address the hydrophilic/hydrophobic interactions between MWCNTs and lipid membranes and the subsequent mechanical properties changes of the systems. In order to elucidate this interaction, we made the following chemical modifications on MWCNTs: oxidized MWCNTs (ox-MWCNTs) displaying reduced hydrophobic surface character, pristine MWCNTs (p-MWCNTs), and alkyl functionalized MWCNTs (alk-MWCNTs) exhibiting enhanced hydrophobic surface properties, were put in contact with GUVs and observed by confocal microscopy. Our observations revealed that the interaction between the CNTs and GUVs depends on the type of chemical functionalization: ox-MWCNTs remain at the membrane interacting with the polar head of the phospholipids, p-MWCNTs internalize GUVs spontaneously, and alk-MWCNTs persist inside the membrane. The mechanical properties of **MWCNTs@GUVs** systems were measured using the electrodeformation method, which shows an increased bending stiffness (**κ**) of the GUVs as MWCNTs concentration increases. High concentrations of p-MWCNTs and alk-MWCNTs induced vesicle adhesion; p-MWCNTs produced a considerable reduction in the average size of the GUVs, while alk-MWCNTs form complex stable structures inside the membrane. The statistical analyses of the experimental results are compared with available computer simulations. The picture emerging from our results is that the interaction between GUVs and MWCNTs is due mainly to hydrophobicity.

## Introduction

The most promising features of carbon nanotubes (CNTs) to bring new exciting functions to biomedical applications are *i*) their ability to penetrate membrane cells^1^. *ii*) their electrical conductivity and morphology that integrate with nervous^2^ and muscular^3^ tissues. *iii*) their high surface area that makes them ideal vehicles for the construction of targeted drug delivery systems^4^; and *iv*) their tunable photophysical properties ideal for the development of biological probes^5^. Although CNTs hold these properties together, the achievement of industrial biotechnological applications appears so far out of reach. There are some unresolved questions on how CNTs interface cells, which reveal an urgent necessity for rigorous research on the physical and chemical interactions that stimulate a biological response. Currently, a considerable amount of experimental information dealing with biomedical applications of CNTs is available^6^. However, controversy exists on experimental results, suggesting that such information is still incomplete. In some cases, CNTs are reported to be excellent intracellular delivery vectors for some drugs^7^ while in other conditions CNTs are found cytotoxic^8^. Unfortunately, available results are difficult to compare among them since the many variables involved vary from experiment to experiment. Also, the synthesis of CNTs often constrains the reproducibility of experimental protocols^6^.

The simplification of cellular systems is an outstanding tool to explore the biophysical bases that govern the interactions of low dimensional materials with cells^9^. The lipid bilayer established as the universal basis for the cell membrane structure is the first barrier that CNTs encounter while interfacing cells. A lipid bilayer has other characteristics besides its self-assembly properties, for instance, its fluidity and deformability, which are crucial to many membrane functions^10^. In the present work, we used giant unilamellar vesicles (GUVs) as a model for cellular membrane systems^11^, interacting with multi-walled carbon nanotubes (MWCNTs) owning different surface properties at different concentrations: oxidized MWCNTs (ox-MWCNTs) carrying hydrophilic oxidized carbon atoms; pristine MWCNTs (p-MWCNTs); and alkyl functionalized MWCNTs (alk-MWCNTs) having hydrophobic alkyl carbon chains covalently attached to its carbon skeleton^12^. MWCNTs interaction with GUVs (**MWCNTs@GUVs**) was followed by phase contrast, fluorescence, and confocal microscopy. MWCNTs were chosen ought to a large number of scientific reports dealing with biomedical and biotechnological applications. Correspondingly, the functionalization and characterization of MWCNTs are more standardized protocols than those of SWCNTs^13^. Furthermore, in the past it has been reported that MWCNTs present lower cytotoxicity than SWCNTs, making them more suitable for future medical applications^14^. It is well known that the mechanical properties of cell membranes change with modifications on the chemical and physical environment^15^, demonstrating that the membrane bending and stretching are essential for cellular functions^16^. To characterize the effect of MWCNTs on the membrane mechanical properties, we measured the bending stiffness of **MWCNTs@GUVs** and compared it with that of GUVs by using the electrodeformation method^17–19^. Briefly, AC electric fields were applied to the **MWCNTs@GUVs** systems, deforming the spheroidal vesicle. The net deformations were measured and used to calculate the bending stiffness (**κ**) of the vesicles^17,18^. Our results show that GUVs display higher **κ** values as MWCNTs concentration increases. At high concentration, p-MWCNTs and alk-MWCNTs extract a high amount of lipids from the membrane producing a tension-induced vesicle adhesion; p-MWCNTs considerably reduced the GUVs size while alk-MWCNTs form complex structures within the membrane. Briefly, ox-MWCNTs interact with the membrane; p-MWCNTs translocate GUVs membranes spontaneously, and alk-MWCNTs remain inside the membrane forming complex stable structures. This study aims to set the basis for the systematic study of the complex interaction between functionalized CNTs and cells. We focused on the simplest interactions, *i.e*., hydrophilic and hydrophobic effects that guide the molecular recognition between the systems. The precise control over these variables is paramount for the development of advanced biomedical applications of carbon nanostructures without risking human health.

## Model System

GUVs provide simple model systems for cell membranes allowing the development of experimental protocols under controlled conditions, as well as the implementation of computer simulations in self-consistent systems. In this work, GUVs were used as a model system to elucidate the main variables guiding the internalization of MWCNTs in cellular membranes. Experiments *in silico* have correlated CNTs-cell translocation to the hydrophobic forces that guide them to internalize the lipid bilayer^13^. Different translocation mechanisms have been proposed depending on the diameter, length, and chirality of CNTs^20^. Experimentally, biomolecules and drugs have been attached to CNTs modifying their surface properties, facilitating membrane translocation^21^.

**Figure 1:**
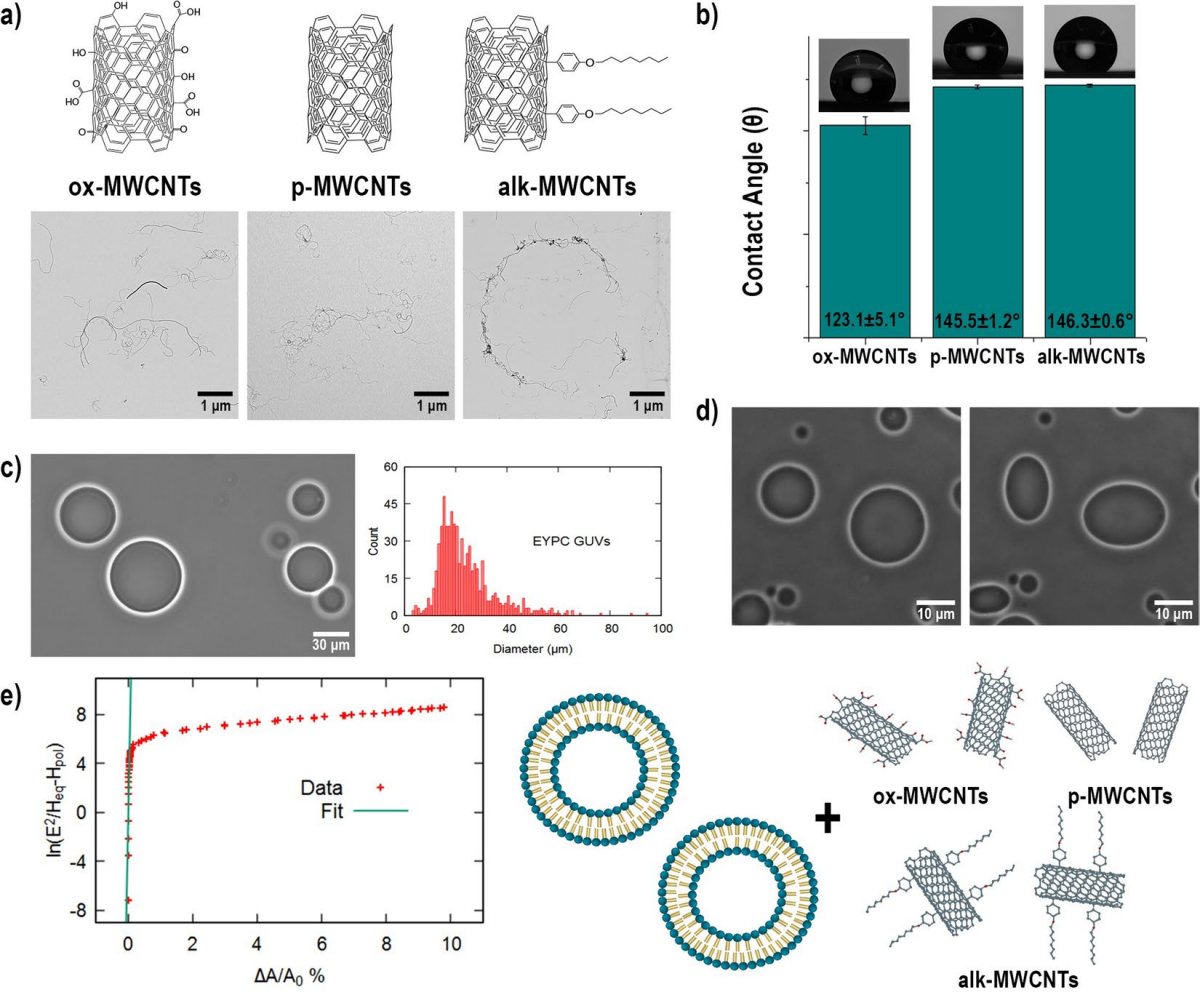
(**a**) Schematic representation and representative TEM images of ox-MWCNTs, p-MWCNTs, and alk-MWCNTs. (**b**) Contact angle (CA) measurements of MWCNTs films produced by vacuum filtration. (**c**) Optical image and diameter histogram of as produced GUVs. ImageJ was used to binarize the images and measure the diameter of each vesicle, for each sample we counted 700 vesicles from which we obtained the size distribution. (**d**) Representative image of GUVs deformation under AC electric fields at 0 V and 10 V. (**e**) Linear fit of the bending regime of GUVs deformation. The slope (green line) of the bending regime domain gives **κ** = 4.5 ± 1.3 × 10^−19^ J. (**f**) Cartoon representing the model system, GUVs, and MWCNTs.

Motivated by previous results, we have chemically functionalized pristine MWCNTs (p-MWCNTs, 9.5 nm diameter, and 1.5 µm length) to attain tubes with hydrophilic and hydrophobic surface character. Oxidation in piranha solution^22^ was completed producing ox-MWCNTs, hydrophilic tubes. Hydrophobic alk-MWCNTs were synthesized by the covalent attachment of alkyl chains of 8 carbon atoms using a diazonium based arylation reaction^23^. ox-MWCNTs, p-MWCNTs and alk-MWCNTs are schematically displayed in Fig. 1a. XPS analysis and TEM images are shown in the Electronic Supporting Information (ESI). Contact angle (CA) measurements, performed on films produced by filtering dispersions of each type of tubes, confirm the modification of the surface properties of MWCNTs by covalent functionalization. CAs were measured on films supported on glass; the MWCNTs films were produced by filtering the CNT dispersions (0.1 mg/mL) of each type. The measurements were done with 5 µL water droplets at three different locations on each film. It was expected that the CA values for p-MWCNTs and alk-MWCNTs would be similar because the morphology of the CNTs is almost the same. Thus the roughness of the systems is the same, only changing the chemical composition of the CNTs. The chemical structure of alk-MWCNTs@GUVs with the carbon chains (8 C atoms) covalently attached to the carbon frame induces a molecular interaction with the hydrophobic alkyl tails of the lipids (16–18 C atoms). We obtained CA values of 123.1 ± 5.1°, 145.5 ± 1.2°, and 146.3 ± 0.6° for ox-MWCNTs, p-MWCNTs, and alk-MWCNTs respectively, results are summarized in Fig. 1b.

GUVs are formed by the electroswelling method^24^ displaying a diameter dispersion ranging from 10–60 µm, observed in Fig. 1c. The changes in the mechanical properties of the GUVs in **MWCNTs@GUVs** systems were measured by the electrodeformation method^17,18^. GUVs containing sucrose solution were dispersed in a glucose medium creating a refractive index mismatch and a density gradient allowing the easy tracking of the systems by optical microscopy techniques. For phase contrast and confocal microscopy analyses, GUVs were exposed to three concentrations of MWCNTs (0.1, 0.5 and 2.0 µg/mL). For the electrodeformation studies, the concentration was reduced to 25, 50 and 75 *n*g/mL since non-deformation of the GUVs was observed at higher concentrations. GUVs were deformed applying an AC electric field at different strength values ranging from 10 to 20,000 V/m. The induced electric stress prompts a change in the apparent area of the GUVs observable by phase contrast microscopy. This process is exemplified in Fig. 1d where typical contrast microscopy images are shown. GUVs are spheroidal in the absence of an external field, which induces the deformation of the vesicle when it is applied. The bending stiffness (**κ**) of GUVs and **MWCNTs@GUVs** systems was calculated using the expression^18^:

1 $$\frac{{\rm{\Delta }}A}{{A}_{0}}=\frac{{k}_{B}T}{8\pi {\boldsymbol{\kappa }}}\,\mathrm{ln}(\frac{{E}_{0}^{2}}{{H}_{po}-{H}_{eq}})+c$$

where A_0_ is the area of the unperturbed spheroidal vesicle, ΔA is the increases of the apparent area, *E*_0_ is the electric field strength, *H* is the main curvature in the equator (*eq*) and pole (*po*) of the vesicle, *T* is the temperature, *k*_B_ is the Boltzmann constant, **κ** is the bending stiffness, and the constant *c* is a frequency dependent factor. The data of deformations, as shown in Fig. 1e exhibits low tensions, known as bending regime, the wrinkles of the GUVs are flattened by the applied electric field. From the slope of the curve in the bending regime, the **κ** value is computed.

After the complete characterization of the main elements of our model, GUVs were incubated with MWCNTs as shown in the schematic representation in Fig. 1f. The spatial localization of the different MWCNTs on GUVs was revealed by the analysis of fluorescence and confocal microscopy of labeled systems. Results allowed us to formulate the possible mechanisms responsible for the assembly of **MWCNTs@GUVs** systems and the changes in the mechanical properties of the GUVs in the presence of MWCNTs. It is important to notice that the systems prepared for confocal microscopy were stabilized using a histological gel while contrast and fluorescence microscopy were measured in aqueous dispersions.

## Results

### ox-MWCNTs@GUVs

**Figure 2:**
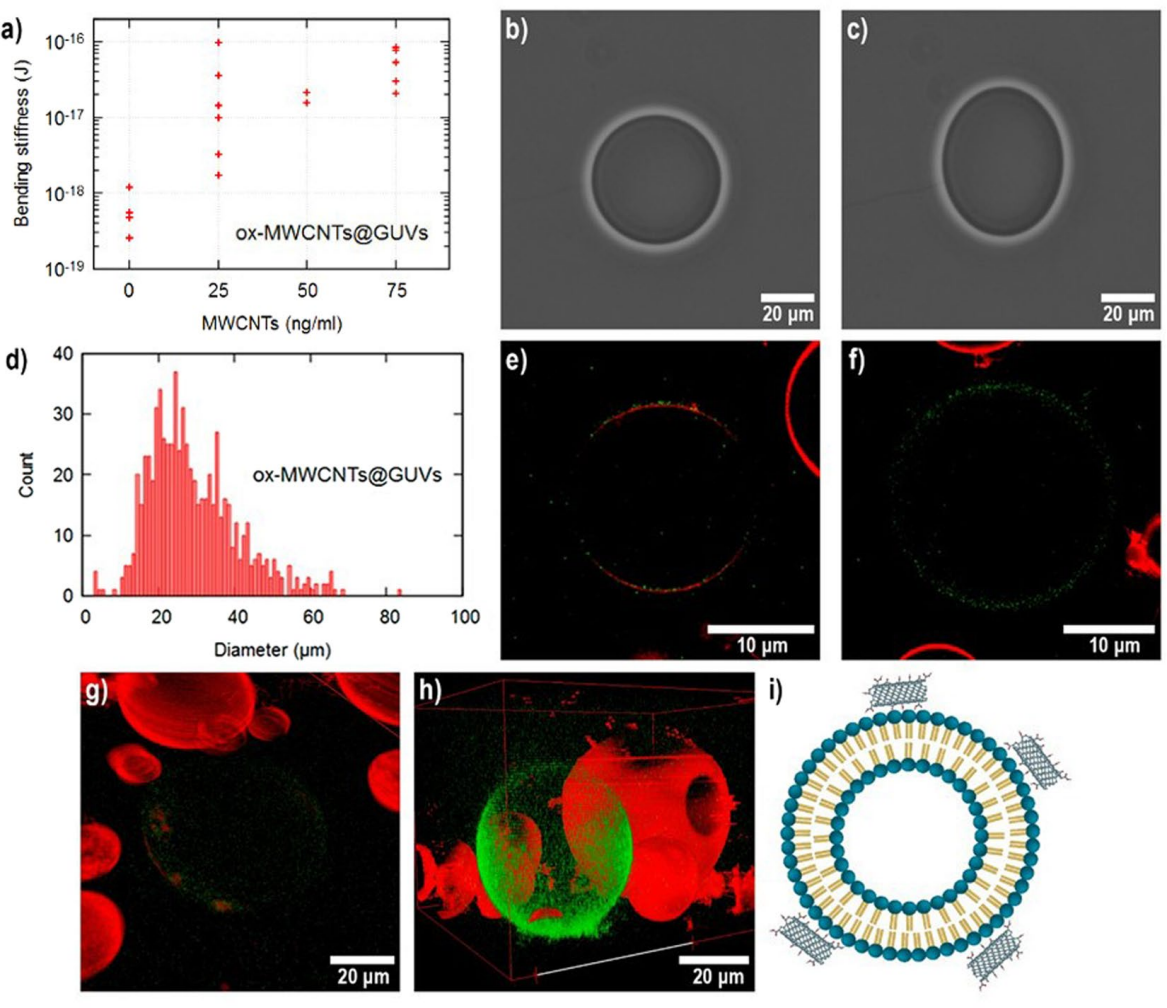
(**a**) Bending stiffness (**κ**) values of individual vesicles at different concentrations of ox-MWCNTs. (**b,c**) **ox-MWCNTs@GUVs** deformation under 0 V/m (**d**) and 20 kV/m; (**d**) Diameter histogram of **ox-MWCNTs@GUVs** at the concentration of 2 µg/mL of CNTs after one hour of incubation. (**e,f**) Confocal images of **ox-MWCNTs@GUVs** at 2 µg/mL. (**g,h**) 3D reconstruction of confocal images of **ox-MWCNTs@GUVs** at a 2 µg/mL. (**i**) Cartoon of the interaction of the system **ox-MWCNTs@GUVs**.

Figure 2 summarizes the characterizations performed on GUVs with ox-MWCNTs. The **κ** values of GUVs exposed to different concentrations of ox-MWCNTs are plotted in Fig. 2a. A clear increase in κ was observed when increasing the tube concentration reaching values as high as 10^4^ K_B_T, three orders of magnitude higher than **κ** values of pure GUVs. Illustrative contrast images of the electrodeformation of the **ox-MWCNTs@GUVs** are shown in Fig. 2b,c. **ox-MWCNTs@GUVs** diameter histogram plotted in Fig. 2d displays a minor modification in the size of **ox-MWCNTs@GUVs** when compare with pure GUVs (Fig. 1c). Confocal microscopy images are shown in Fig. 2e,f revealed that ox-MWCNTs mainly localize on the GUVs surface. The proximity of green-labeled ox-MWCNTs to the red-head polar groups of the lipids induced the quenching of GUVs (Fig. 2e) until only the green fluorescence of ox-MWCNTs was detected (Fig. 2f). It has been reported by molecular dynamics simulations, that phospholipids in the membrane tend to form inverse micelles when interacting with oxygen moieties on the surface of carbon nanostructures as graphene oxide^25^. Thus we propose the formation of inverse micelles as the mechanism responsible for the quenching of GUVs red fluorescence. This rearrangement of phospholipids on the surface of MWCNTs shortens the distance between the lattice structure of MWCNTs and the polar head groups of phospholipids causing the quenching of rhodamine. 3D image reconstruction, Fig. 2g,h clearly shows that ox-MWCNTs are completely covering the GUVs. These observations coincide with reports from calculations indicating that the energy costs for CNTs to cross the membrane is higher than the thermal energy, indicating endocytosis as the most probable mechanism for the translocation of CNTs^26^. The **κ** values of **ox-MWCNTs@GUVs** systems increased considerably with the increase in the concentration of MWCNTs until non-deformation was observed. Thus, from our data, it is evident that an interaction between GUVs and ox-MWCNTs is occurring. We found the following processes as the most probable interaction mechanisms: GUVs are 2D membranes nearly incompressible acting as capacitors formed by the insulator lipid bilayer. When an electric field is applied, the tension induces the electrodeformation of the vesicles^27^. MWCNTs are excellent conductive materials; it is widely accepted that ox-MWCNTs keep their conductivity properties after oxidation since only the external tube is “partially” destroyed maintaining the inner tubes intact. Thus, in **ox-MWCNTs@GUVs** systems, conductive ox-MWCNTs situated at the membrane screen the electric field lines decreasing GUVs deformations. At a high concentration, ox-MWCNTs may act as a Faraday cage hindering electromagnetic fields interaction. Complementary scenery involves the formation of inverse micelles around the ox-MWCNTs due to thermal fluctuations. The formation of inverse micelles might explain the increment in the **κ** values and the red quenching of the GUVs observed by confocal microscopy. A similar mechanism has been previously reported by Lelimousin, *et al*.^28^ SWCNTs of 5 nm diameters and lengths greater than 6.6 nm translocate the membrane in a two-stepwise mechanism. Lipids on the membrane first move to the inner region of the SWCNTs and form a transient water pore stabilized by the interaction of the polar heads of the lipids with the water molecules. After pore formation, SWCNTs are embedded in the membrane tilting parallel to it while the pore disappears. Then the lipids formed an inverse micelle with the remained water molecules trapped in the tube. Although oxidized carbon atoms in ox-MWCNTs on contact with GUVs may facilitate the production of inverse micelles, water molecules interacting with the hydrophilic groups may delay micelle formation. In our experiments, translocation was not observed, however, the accumulation of ox-MWCNTs on the lipid membrane of <2 nm width produced a completely red quenching and an increase in the membrane width observed as a thin green line in Fig. 2f. It was demonstrated by Tu *et al*. that lipid extraction by graphene oxide nanosheets is a robust process^29^. Lipid collective movement initiated by the short-range van der Waals attractions between graphene oxide and lipids, once extracted, the strong hydrophobic interactions between the reminiscence graphene zones and lipids tails played an important role in nanoscale dewetting, expelling the water molecules from the surface of the carbon nanomaterial. Following a similar mechanism as in graphene oxide, inverted lipid micelles should be covering ox-MWCNT interacting with GUVs membrane and positioning the tubes parallel to it. During the oxidation process of CNTs, the tips of the tubes are firstly destroyed as a result of the higher curvature attaining higher oxidation degree, considerably reducing the hydrophobicity and then hindering the perpendicular arrangement of the tubes. It is expected that both the electric field screening and formation of inverted micelles act together to increase **κ** values. Exploration of more complex systems is required to completely elucidate the interaction mechanism, including GUVs formed by charged lipids with adjusted ionic force and pH. A schematic representation of the model system is shown in Fig. 2i.

The above process is similar to the three-dimensional conformation of biomolecules resulting from the interaction of their constituents and water molecules. Dehydration caused an extreme change in the physical properties of biomolecules. It has been experimentally observed that graphene and graphene oxide materials induce the degradation of bacteria cell membranes^29^.

### p-MWCNTs@GUVs

**Figure 3:**
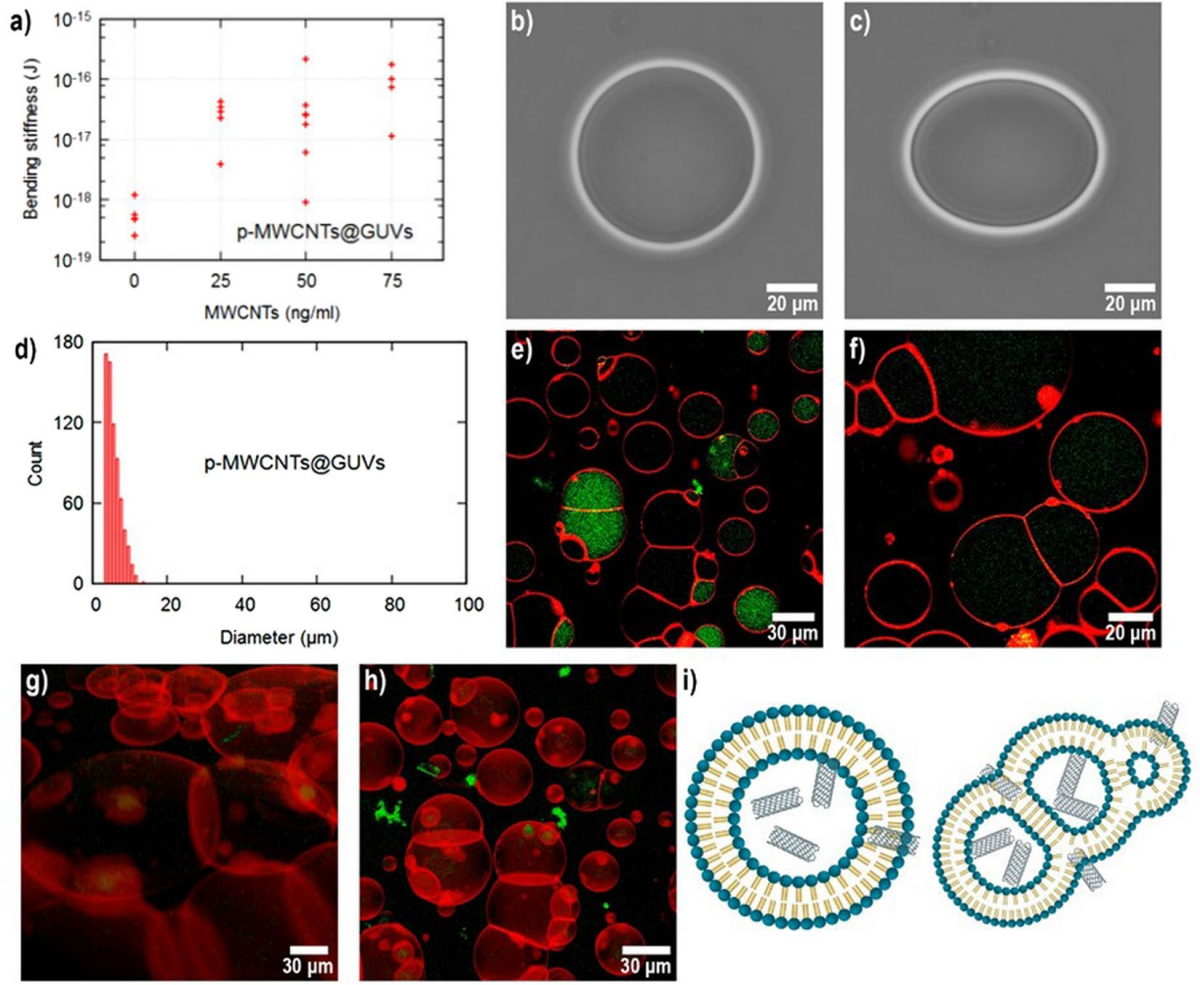
(**a**) Bending stiffness (**κ**) values of individual vesicles at different concentrations of p-MWCNTs. (**b**) **p-MWCNTs@GUVs** deformation under 0 V/m (**c**) and 20 kV/m AC electric fields at a 75 ng/mL CNT concentration. (**d**) Diameter histogram of **p-MWCNTs@GUVs** at a 2 µg/mL concentration of CNTs (**f–i**) Confocal images of **p-MWCNTs@GUVs** at a 2 µg/mL concentration. (**b**) Cartoon of the interaction in the p-**MWCNTs@GUVs** systems.

Figure 3 summarizes the interactions of GUVs with p-MWCNTs. An increase of three orders of magnitude in **κ** values was observed at the highest concentration of p-MWCNTs. When the experiments were performed, erratic behavior was noticed, some GUVs do not deform while other GUVs showed **κ** values similar to pure ones, results are plotted in Fig. 3a. Illustrative contrast images of the electrodeformation of **p-MWCNTs@GUVs** are shown in Fig. 3b,c. **p-MWCNTs@GUVs** diameter histogram shown in Fig. 3d evidently displays a considerable reduction on the GUVs size compared with pure GUVs (Fig. 1c). Confocal microscopy images are shown in Fig. 3e,f revealed p-MWCNTs were inside the GUVs. Interestingly, GUVs were mostly found forming chains up to 12 vesicles. The 3D image reconstruction, shown in Fig. 2g,h revealed p-MWCNTs inside the GUVs forming chains of adhered vesicles. Quenching of fluorescence was not immediately observed as result of **p-MWCNTs@GUVs** interactions. However, green photobleaching was noticed over time in the sample (images available in ESI). From all this evidence, we hypothesize the following mechanisms responsible for the formation of **p-MWCNTs@GUVs** systems: p-MWCNTs interact with the non-polar chains of the lipids, the inner part of the GUVs membrane. This interaction is strong enough to allow GUVs translocation. Full-atom MD computer simulations showed that when 6 nm long (5, 5) SWCNTs were introduced perpendicular to DOPC lipid bilayers, the interactions are strong enough to diminish the self-diffusion coefficient, resulting in the modification of the mechanical properties of the membranes^30^. In a different MD simulation experiment, authors showed that embedded N-DWCNTs in DMPC lipid bilayers reduced the entropy of the lipid molecules, limiting the conformational states adjacent to the tubes, hindering lipid diffusion^31^. These strong hydrophobic interactions could explain the increase of the **κ** values of **p-MWCNTs@GUVs** systems. However, for surpassing the high energetic barriers required for membrane adhesion, a complementary mechanism is required. MD computer simulations showed that CNTs could lower the distinct barriers for merging vesicles to fusion^32^. Following the results presented by the authors, we concluded that the strong van der Waals interaction between GUVs and p-MWCNTs capture the lipids on p-MWCNTs covering its entire outer surface. This process might explain why p-MWCNTs were randomly found inside some GUVs and not on the entire sample, Fig. 3e. The covering of tubes by the lipids might induce green fluorescence quenching during the time; this process can be observed in Fig. 3f and in the ESI. Later, the tails of p-MWCNTs in **p-MWCNTs@GUVs** trap other **p-MWCNTs@GUVs** in the proximity, forming a metastable structure of **p-MWCNTs@GUVs** pierced by p-MWCNTs. Additionally, the penetration of p-MWCNTs increases the viscosity of the GUVs internal medium reducing the capacity of membrane displacement due to an external force diminishing deformability. This process does not increase the **κ** values but increases the pressure over the membrane due to a denser medium obstructing the relaxation process. Then, lipids are rearranged in the presence of p-MWCNTs in smaller vesicles adhered to each other. Our results are in complete agreement with computer simulations presented in ref.^32^. In the absence of p-MWCNTs, vesicles at close contact for a long period of times did not adhere, meanwhile, in the presence of p-MWCNTs we found small GUVs adhered in the form of vesicle chains. P-MWCNTs may cause the poration of vesicles promoting vesicle fusion. Hence the difference in the size distribution from GUVs without p-MWCNTs. A schematic representation of the model system is shown in Fig. 3i.

These results are significant since cell adhesion organizes the structures of tissues mediating mechanical, chemical and electrical integration with the surroundings. Cell adhesion is an energy consuming process facilitated by molecules in the cell membrane such as selectins, integrins, syndecans, and cadherins essential for multicellular structure, signal transduction, immune response and protozoan pathogenic mechanisms such as malaria^33^. In artificial membranes, adhesion is performed by sticker molecules, like negatively or positively charged lipids, polysaccharides, etc^34^.

### alk-MWCNTs@GUVs

**Figure 4:**
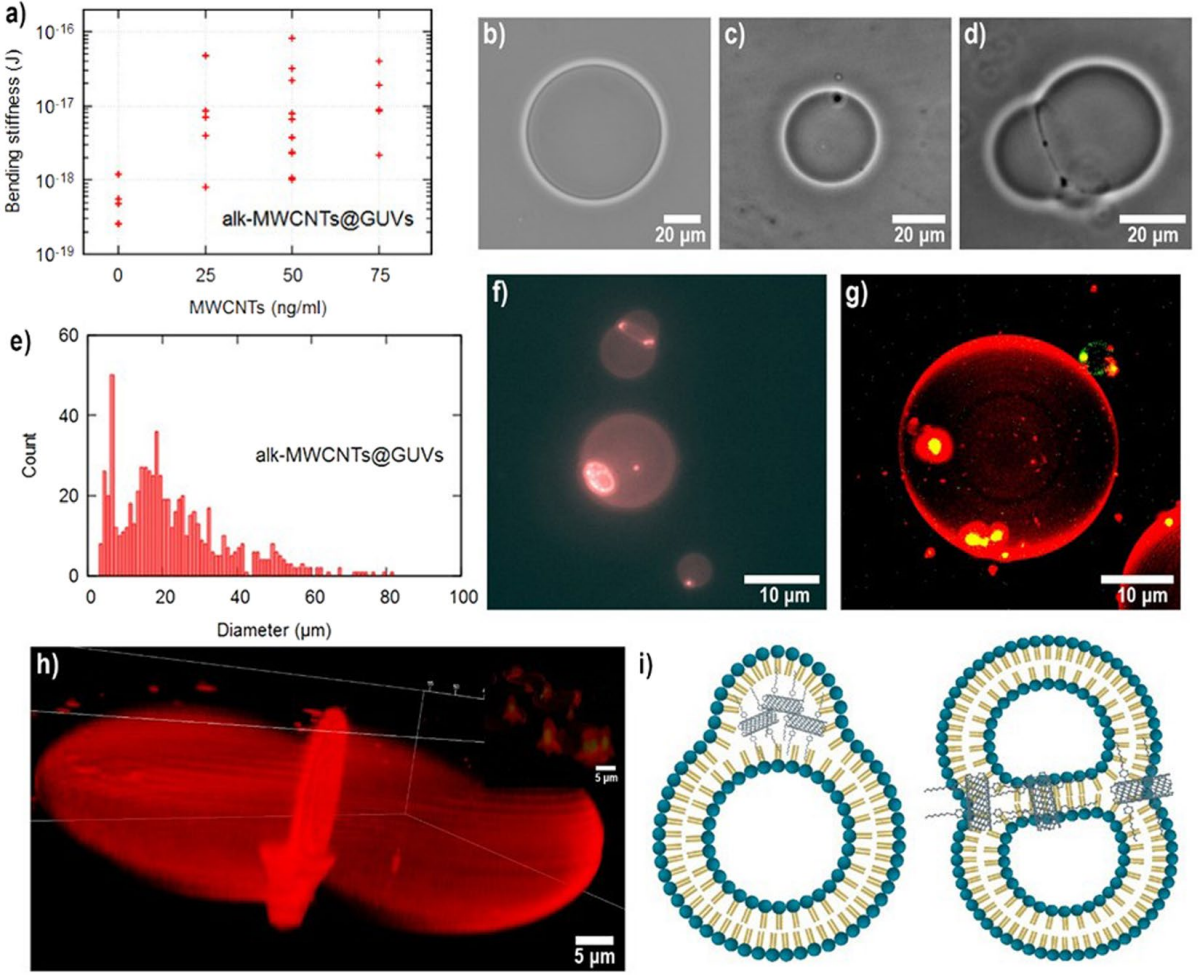
(**a**) Bending stiffness (**κ**) values of individual vesicles at different concentrations of alk-MWCNTs. Phase contrast microscopy images of **alk-MWCNTs@GUVs** at (**b**) 0 µg/mL (**c**), 0.5 µg/mL (**d**) and 2 µg/mL alk-MWCNTs. (**e**) Diameter histogram of **alk-MWCNTs@GUVs** at a 2 µg/mL concentration of alk-MWCNTs. (**f**) Fluorescence image of **alk-MWCNTs@GUVs**. (**g**) Confocal images of **alk-MWCNTs@GUVs** at a 2 µg/mL CNTs concentration, colocalization of alk-MWCNTs GUVs on “ghost-like” structures. (**h**) 3D reconstruction of a GUV including the “ghost-like” structure. (**i**) Cartoon of the interaction in the **alk-MWCNTs@GUVs** system.

Figure 4 summarizes the interactions of GUVs with alk-MWCNTs. As for the systems described above, there is also an increase in the **κ** values of **alk-MWCNTs@GUVs**, results are plotted in Fig. 4a. From the graph, erratic behavior is noticed, similar to the one observed for **p-MWCNTs@GUVs**. Interestingly, the analysis of images obtained by contrast microscopy reveals the formation of new structures. In Fig. 4b a typical GUVs image is shown, Fig. 4c shows an image of the **alk-MWCNTs@GUVs** system at a 0.5 µg/mL alk-MWCNTs concentration. The image exposes a black spot on the membrane surface. The spots freely diffuse over the GUVs surface while the vesicle is entirely stable. Surprisingly, when the concentration of the tubes was increased to 2 µg/mL, the number of black spots increases enhancing vesicle adhesion, Fig. 4d. The **alk-MWCNTs@GUVs** size histogram plotted in Fig. 4e shows a reduction of the GUVs size with an extensive distribution compared with p-MWCNTs. The black spots observed by contrast microscopy were revealed out as brighter reddish structures by fluorescent microscopy, shown in Fig. 4f. The increase in the fluorescence intensity on specific zones indicates lipid accumulation on the alk-MWCNTs. Confocal microscopy clearly shows that alk-MWCNTs are trapped in the inner part of the membrane as shown in yellow, indicative of close interactions between green labeled alk-MWCNT and red marked lipid polar heads. The 3D image reconstruction, shown in Fig. 4i displays the formation of a complex hybrid “ghost-shape” structure. It is challenging to formulate a straightforward mechanism for the formation of **alk-MWCNTs@GUVs** systems. The “ghost-shaped” aggregates formed inside the lipid membrane appear to be stable hybrid structures since several objects with the same shape were found to persist throughout the sample, see Fig. 4h. As a first attempt to explain the formation mechanism of **alk-MWCNTs@GUVs** systems, we propose that additionally to the hydrophobic forces that guide p-MWCNTs to translocate GUVs membranes, the alkyl chains (8 C atoms) covalently attached to the alk-MWCNTs interact with the hydrophobic alkyl tails of the lipids (16–18 C atoms). X-ray scattering (SAXS) experiments have demonstrated that membrane affinity and penetration depth of molecules possessing alkyl chains in lipid membranes increases depending on the alkyl chain length until a threshold is reached^35^. Thus, alk-MWCNTs enter the lipid membrane guided by hydrophobic forces and remain trapped between the lipids’ tails as a result of the alkyl chains interaction. The thermal motion allows alk-MWCNTs to freely diffuse inside the membrane bundling as tube concentration increases forming “ghost-shaped” aggregates. We propose that lipid chains adopt ordered conformations in the vicinity of the MWCNTs by tail-tail recognition. Thus, the energy of deformations and the entropic conformational changes of the GUV-MWCNT systems lead to energetic-entropic rearrangements of the lipids. Once an aggregate reaches a threshold size, another one is formed, most probably as a result of energy-entropic processes. Then, tension induced vesicle adhesion occurs, presumably, between GUVs with aggregates already formed since most of the contact areas between adhered vesicles are delimitated by “ghost-shaped” aggregates, see Fig. 4h.

## Discussion and Conclusions

MWCNTs have been proposed to be used for different biomedical applications. For example, DNA-encasement of MWCNTs results in well dispersed in water Polyethylenimine (PEI) amino functionalized SWNTs and MWCNTs to deliver siRNAs into HeLa-S3 cells. The amount of grafted PEI was higher in MWCNTs as they do not aggregate as much as SWCNTs^36^. Also, scaffolds composed of MWCNTs and chitosan have been shown to be biocompatible and biodegradable, promoting cell adhesion, viability, and proliferation^37^.

MWCNTs make excellent scaffolds to synthesize new intelligent devices for biomedical applications, because they have more standardized methods of chemical functionalization, can penetrate the membrane of mammalian cells, exhibit lower cytotoxicity than SWCNTs, and also present a large surface area which allows the attachment of different biomolecular groups (such as nucleic acids, peptides, proteins, and drugs^37^) to their surface, granting the opportunity to prepare tailored devices^38^.

In our experiments, we observed that the interaction between CNTs and GUVs is dependent on the type of surface chemical functionalization present on the MWCNTs. ox-MWCNTs interact with the external membrane of the GUVs, extracting lipids from the vesicles to reduce the interaction of the non-functionalized patches on the surface of ox-WMCNTs. This effect can promote the formation of pores in the vesicle surface. If a small pore is formed, the auto-assemble of the vesicle keeps their shape as is observed in pore formation experiments^39^. Because ox-MWCNTs might be promoting pore formation in GUVs, our results might have implications on the design of multifunctional drugs to kill bacteria and simultaneously neutralize toxins released by bacteriolysis by molecular dehydration. p-MWCNTs translocate the GUVs membranes because of their hydrophobicity. At higher concentrations, p-MWCNTs destabilize the GUVs surface causing membrane adhesion. The excellent control of the energetic and entropic mechanism for membrane adhesion by p-MWCNTs might find applications in biomedicine, by producing new drug delivery vehicles or functional surfaces^40^ that enable the rapid, efficient, and tunable cell adhesion independent of biomolecules. Finally, because of the functional groups attached to alk-MWCNTs, this CNTs could mimic biological processes such as the ones performed by bacteriophages, in which protein spikes are translocated through the lipid bilayer without undergoing denaturalization^41^. The mimicking of cellular functions may profoundly impact the development of new therapies for diseases such as cancer and genetic disorders. The recent progress in supramolecular and dynamic chemistry triggers exciting opportunities for the simple design of reversible ensembles for the construction of functional CNTs-lipid membrane assemblies allowing the design of safe biomedical applications.

The surface properties of MWCNTs are crucial while interfacing lipid membranes. At low concentration of MWCNTs, the mechanical properties of GUVs are preserved, however, at a concentration as lower as 75 *n*g/mL, the **κ** of the GUVs is compromised by the presence of MWCNTs regardless of their surface properties. The **κ** value increases as a result of a higher concentration of MWCNTs, and there is not a tendency toward MWCNTs functionalization. The local energetic-entropic rearrangement of the lipids on the tube depends on their superficial characteristics. The interaction between lipids and MWCNTs induce bilayer deformations with an energy cost. Oxidized carbon atoms acting as hydrophobic functional groups on ox-MWCNTs induce the formation of inverse micelles on the tube surface. The decrease on the hydrophobic properties of ox-MWCNTs obstructs membrane translocation, positioning the tubes parallel to the GUVs. The strong hydrophobic forces between p-MWCNTs and lipids allow membrane translocation by the complete covering of p-MWCNTs by lipids. Finally, the interaction between alk-MWCNTs and the tails of the lipids in the GUV membrane induces the formation of complex stable “ghost-shaped” structures. For all the cases, van der Waals forces guide the interaction between MWCNTs and GUVs, while strong hydrophobic forces are responsible for membrane translocation.

Our results in combination with recent advances in supramolecular and dynamic chemistry might pave the way for the tailored synthesis of new advanced complex systems for multifunctional drugs to kill bacteria and simultaneously neutralize toxins released by bacteriolysis, drug delivery vehicles, the fabrication of functional surfaces for cell growth and adhesion and new therapies for diseases, such as cancer and genetic disorders.

## Materials and Methods

Lipids were purchased from Avanti Lipids. L-α-phosphatidylcholine (EggPC, #840051) and fluorescent dye L-α phosphatidylethanol-amine N-(lissamine rhodamine B sulfonyl) (Egg-Rh PE, #810146) were used from stock solutions of 4 mg/ml in chloroform. Thin-MWCNTs were purchased from NANOCYL NC7000 (www.nanocyl.com). All chemicals and solvents were purchased from Sigma-Aldrich and used without further purification.

### Experimental procedures

#### ox-MWCNTs

Pristine MWCNTs (p-MWCNTs) were oxidized using a piranha solution (70:30, 96 wt% H_2_SO_4_, 30 wt% H_2_O_2_), following the method described by Galiotis and cools^22^. 30 mg of p-MWCNTs were dispersed in 60 ml of a piranha solution and refluxed for 5 hours at 70 °C under magnetic stirring. The sample was neutralized, filtered and washed exhaustively with deionized water. After that, ox-MWCNTs were dispersed in distilled water.

#### alk-MWCNTs

p-MWCNTs were covalently functionalized with 4-octyl-oxianiline using the diazonium based arylation reaction^12^. For this, 15 mg of p-MWCNTs were dispersed in 60 ml of distilled water and sonicated for 20 min using an ultrasonic bath. Then, 4 eq. of 4 octyloxianiline and 3 eq. of isoamyl nitrite were added to the mixture. The dispersion was put under magnetic stirring at 80 °C overnight. After that, the mixture was filtered and washed thoroughly with DMF, methanol, and chloroform. Finally, functionalized alk-MWCNTs were dispersed in chloroform.

The chemical composition of the samples was studied by X-ray Photoelectron Spectroscopy (XPS) using a Versaprobe PHI 5000 spectrometer from Physical Electronics, equipped with a monochromatic Al K α X-ray source with a 0.7 eV energy resolution. Results are reported in ESI. The morphology of p-MWCNTs and f-MWCNTs was analyzed using a TEM JEM-JEOL-2100 at 200 kV. Samples were prepared by drop casting of MWCNTs dispersions. Images are reported in ESI.

### Fluorescent MWCNTs

ox-MWCNTs, p-MWCNT, and alk-MWCNT were non-covalently functionalized with fluorescein isothiocyanate (FITC) by mixing 1 mg of FITC, and 1 mg of MWCNTs in 5 ml of Milli Q water^42^. Dispersions were sonicated for 30 min using an ultrasonic bath and then kept in the complete dark under magnetic stirring at room temperature overnight. The non-covalent functionalization achieved by sonication allows π-π interaction between FITC and the carbon lattice. The hydrophobic aromatic fluorescein group bind to the sidewall of CNTs by π stacking. It has been reported that this type of functionalized materials is stable at neutral pH^42^. Fluorescence MWCNTs were filtered and washed thoroughly with Milli Q water until no absorption of FITC was detected in the filtered solution by UV-Vis spectroscopy. Fluorescent MWCNTs were used as prepared.

### Electroswelling

GUVs were prepared using a modified version of the electroformation method reported by Angelova, and Dimitrov^24^. 4 μL of 4 mg/mL of the lipid solution was spread on the conductive surface of two indium tin oxide-coated glass plates and kept at room temperature under vacuum for approximately 1.5 h to remove all traces of the organic solvent. The two glasses were placed with their conductive sides facing each other and separated by a 1 mm thick Teflon frame to form a chamber, which was sealed with silicon grease. The chamber was filled with a 0.2 M sucrose solution. The glass plates were connected to a function generator and an alternating current, frequency = 10 Hz was applied varying intensities starting at 1 V_pp_ for 15 min, then increased to 1.5 V_pp_ for another 15 min and lastly increased to 2 V_pp_ for 1.5 h. Then, to detach the GUVs from the glass surfaces an alternating current of 3 Hz and 2 V_pp_ was applied for half an hour. The GUVs solution was removed from the electroswelling chamber and stored at 4 °C.

### Confocal Microscopy

For the visualization of the interaction of MWCNTs@GUVs a confocal microscope LSM 710 NLO Carl Zeiss was used operating with excitation wavelengths of 488 and 561 nm with a Plan-Apochromat 63 × −1.40 oil DIC M27 objective. MWCNTs@GUVs were reconstructed with dimensions of 224.48 µm × 224.48 µm with z sections of 0.849 µm. Samples were prepared by diluting 20 µL of the initial GUVs dispersion in 1 ml of 0.2 M glucose solution. Later, 20 µL of 0.1 mg/ml of MWCNTs dispersion was added to the diluted dispersion of GUVs resulting in a final concentration of 2.0 µg/ml of MWCNTs; the mixture was incubated for 30 minutes at room temperature. The chamber for the sample visualization was sealed with a gelatin-coating solution.

### Electrodeformation

To evaluate the changes in the mechanical properties of MWCNTs@GUVs the electrodeformation of the systems was performed. The deformation experiments were followed using a Leica LED MD phase contrast microscope (Leica Microsystems Heidelberg GmbH, Germany) with a 40x Ph2 objective. The deformation assays were performed in an electrodeformation chamber which consisted of two parallel cylindrical electrodes separated by a 500 µm gap purchased from Eppendorf. GUVs and **MWCNTs@GUVs** systems were prepared by diluting 26 µL of the initial GUVs dispersion with 3970 µL of 0.21 M glucose solution. In different sets of experiments, 4 µL of a 0.1 mg/ml MWCNTs dispersion was added to diluted GUVs. Mixtures were then incubated for one hour in gentle agitation (50 rpm). **MWCNTs@GUVs** systems were electrodeformed by applying an alternating electric field of 100 kHz frequency and varying strength ranging from 0 to 20 kV/m. **MWCNTs@GUVs** images were acquired using a Leica DMC2900 camera. The images were processed with ImageJ to extract the principal axes of the vesicles. The homemade software was used to calculate the GUVs surface tension and the changes in the apparent surface area of GUVs.

### Experimental data analysis

#### TEM MWCNTs length and diameter measurement

The lengths and diameters of the modified MWCNTs were obtained by analyzing TEM images of the samples. Using ImageJ software, 30 individual carbon nanotubes were measured in order to obtain the average length and diameter. Here we report the average lengths, diameters and standard deviation for the modified MWCNTs, see Table 1.

**Table 1:** Lengths and diameters of modified MWCNTs obtained by analyzing TEM images.

	Length (µm)	SD	Diameter (nm)	SD
ox-MWCNTs	1.10	0.49	9.92	2.03
alk-MWCNTs	1.13	0.79	9.6	2.37

No significant changes in the dimension parameters were observed after MWCNT chemical modification. Pristine MWCNTs were purchased from NANOCYL, reporting an average length of 1.5 µm and an average diameter of 9.5 nm for the CNTS.

### Bending stiffness

Using Eq. 1, it is possible to estimate the bending stiffness of each vesicle by measuring the length of the major and minor axis of the GUVs during the deformation process. Each measurement has a two-pixel deviation (0.29 µm); this gives us ±1.4% in the area changes of the vesicle deformation. We use Origin to estimate the slope of Eq. 1 to obtain the bending stiffness of individual vesicles. We compute the mean and standard deviation of the experiments using the bending stiffness calculated for each independent vesicle.

### Contact Angle

To estimate the surface properties of the MWCNTs, we measured their contact angles (CAs) using a Ramé-Hart NRL C.A. goniometer, model 295-E1. CAs were measured on films supported on glass; the MWCNTs films were produced by filtering the CNT dispersions (0.1 mg/mL) of each type.

The measurements were done with 5 µL water droplets at three different locations on each film. All measurements were done at room temperature, and the reported CA is the mean value of the 100 data points obtained during the experiments. The error bars in Fig. 1. represents the standard deviation of the mean value of the experiments.

## Electronic supplementary material

XPS, TEM, and Confocal Images.

Ghost-like over GUVs surface

Ghost-like over GUVs surface_2

## Data Availability

All data generated and/or analyzed during this study is available from the corresponding authors on reasonable request.

## References

[1] Kostarelos K (2007). Cellular uptake of functionalized carbon nanotubes is independent of functional group and cell type. Nat. Nanotech..

[2] Malarkey EB (2009). Conductive Single-Walled Carbon Nanotube Substrates Modulate Neuronal Growth. Nano Lett..

[3] Shin SR (2013). Carbon-Nanotube-Embedded Hydrogel Sheets for Engineering Cardiac Constructs and Bioactuators. ACS Nano.

[4] Lacerda L, Bianco A, Prato M, Kostarelos K (2006). Carbon nanotubes as nanomedicines: From toxicology to pharmacology. Adv. Drug Deliv. Rev..

[5] Kouklin NA, Kim WE, Lazareck AD, Xu JM (2005). Carbon Nanotube Probes for Single-Cell Experimentation and Assays. Appl. Phys. Lett..

[6] Serpell CJ, Kostarelos K, Davis BG (2016). Can Carbon Nanotubes Deliver on Their Promise in Biology? Harnessing Unique Properties for Unparalleled Applications. ACS Cent. Sci..

[7] Bianco A, Kostarelos K, Prato M (2005). Applications of carbon nanotubes in drug delivery. Curr. Opin. Chem. Biol..

[8] Lam C-W, James JT, McCluskey R, Arepalli S, Hunter RL (2006). A Review of Carbon Nanotube Toxicity and Assessment of Potential Occupational and Environmental Health Risks. Crit. Rev. Toxicol..

[9] Lipowsky R (1991). The conformation of membranes. Nature.

[10] Ken Jacobson K, Mouritsen OG, Anderson RGW (2007). Lipid rafts: at a crossroad between cell biology and physics. Nat. Cell Biol..

[11] Dimova R (2006). A practical guide to giant vesicles. Probing the membrane nanoregime via optical microscopy. J. Phys.: Condens. Matter..

[12] Mata-Cruz I (2017). Mimicking rose petal wettability by chemical modification of graphene films. Carbon.

[13] Baoukina S, Monticelli L, Tieleman DP (2013). Interaction of Pristine and Functionalized Carbon Nanotubes with Lipid Membranes. J. Phys. Chem. B..

[14] Jia G (2005). Cytotoxicity of Carbon Nanomaterials: Single-Wall Nanotube, Multi-Wall Nanotube, and Fullerene. Environ. Sci. Technol..

[15] Stevens MM, George JH (2005). Exploring and Engineering the Cell Surface. Interface. Science.

[16] Keren K (2011). Cell motility: the integrating role of the plasma membrane. Eur. Biophys. J..

[17] Kummrow M, Helfrich W (1991). Deformation of giant lipid vesicles by electric fields. Phys. Rev. A..

[18] Frickel N, Dimova R (2016). GM1 Softens POPC Membranes and Induces the Formation of Micron-Sized Domains. Biophys. J..

[19] Gracia RS, Bezlyepkina N, Knorr RL, Lipowsky R, Dimova R (2010). Effect of cholesterol on the rigidity of saturated and unsaturated membranes: fluctuation and electrodeformation analysis of giant vesicles. Soft Matter.

[20] Skandani AA, Zeineldin R, Al-Haik M (2012). Effect of Chirality and Length on the Penetrability of Single-Walled Carbon Nanotubes into Lipid Bilayer Cell Membranes. Langmuir.

[21] Klumpp C, Kostarelos K, Prato M, Bianco A (2006). Functionalized carbon nanotubes as emerging nanovector for the delivery of therapeutics. BBA Biomemb..

[22] Datsyuk V (2008). Chemical oxidation of multiwalled carbon nanotubes. Carbon.

[23] Quintana M, Prato M (2009). Supramolecular Aggregation of Functionalized Carbon Nanotubes. Chem. Commun..

[24] Angelova MI, Dimitrov DS (1986). Liposome Electroformation. Faraday Discuss..

[25] Mao J, Guo R, Yang L-T (2014). Simulation and analysis of cellular internalization pathways and membrane perturbation for graphene nanosheets. Biomaterials..

[26] Pogodin S, Baulin VA (2010). Can a Carbon Nanotube Pierce through a Phospholipid Bilayer?. ACS Nano..

[27] Dimova R, Lipowsky R (2017). Giant Vesicles Exposed to Aqueous Two-Phase Systems: Membrane Wetting, Budding Processes, and Spontaneous Tubulation. Adv. Mater. Interf..

[28] Lelimousin M, Sansom MS (2013). Membrane perturbation by carbon nanotube insertion: pathways to internalization. Small.

[29] Tu Y (2013). Destructive extraction of phospholipids from Escherichia coli membranes by graphene nanosheets. Nat. Nanotech..

[30] Parthasarathi R, Tummala NR, Striolo A (2012). Embedded Single-Walled Carbon Nanotubes Locally Perturb DOPC Phospholipid Bilayers. J. Phys. Chem. B..

[31] Li X, Shi Y, Miao B, Zhao Y (2012). Effects of Embedded Carbon Nanotube on Properties of Biomembrane. J. Phys. Chem. B..

[32] Bhaskara RM, Linker SM, Vögele M, Köfinger J, Hummer G (2017). Carbon Nanotubes Mediate Fusion of Lipid Vesicles. ACS Nano.

[33] Gumbiner BM (1996). Cell Adhesion: The Molecular Basis of Tissue Architecture and Morphogenesis. Cell..

[34] Weikl TR, Lipowsky R (2006). Chapter 4, Membrane Adhesion and Domain Formation. Adv. Planar Lipid Bilayer Liposomes.

[35] Durand E (2017). The nonlinear effect of alkyl chain length in the membrane interactions of phenolipids: Evidence by X-ray diffraction analysis. Eur. J. Lipid Sci. Technol..

[36] Huang YY, Terentjev EM (2012). Dispersion of Carbon Nanotubes: Mixing, Sonication, Stabilization, and Composite Properties. Polymers.

[37] Abarrategi A (2008). Multiwall carbon nanotube scaffolds for tissue engineering purposes. Biomaterials.

[38] Wong N, Kam S, Dai H (2005). Carbon Nanotubes as Intracellular Protein Transporters: Generality and Biological Functionality. J. Am. Chem. Soc..

[39] Moroz JD, Nelson P (1997). Dynamically stabilized pores in bilayer membranes. Biophys. J..

[40] Cellot G (2011). Carbon Nanotube Scaffolds Tune Synaptic Strength in Cultured Neural Circuits: Novel Frontiers in Nanomaterial-Tissue Interactions. J. Neurosci..

[41] Browning C, Schneider MM, Bowman VD, Schwarzer D, Leiman PG (2012). Phage Pierces the Host Cell Membrane with the Iron-Loaded Spike. Cell Press.

[42] Nakamaya-Ratchford N, Bangsaruntip S, Sun X, Welsher K, Dai H (2007). Noncovalent Functionalization of Carbon Nanotubes by Fluorescein-Polyethylene Glycol: Supra-molecular Conjugates with pH-Dependent Absorbance and Fluorescence. J. Am. Chem. Soc..

