# Supplementary material for: Interactions of Functionalized Multi-Wall Carbon Nanotubes with Giant Phospholipid Vesicles as Model Cellular Membrane System
Source: Sci Rep. 2018 Dec 20;8:17998. doi: 10.1038/s41598-018-36531-9 (PMC6302088; doi:10.1038/s41598-018-36531-9)
Supplement: Supplementary file 1 — XPS, TEM, and Confocal Images. [file 41598_2018_36531_MOESM1_ESM.docx]

**Interactions of Functionalized Multi-Wall Carbon Nanotubes with Giant Phospholipid Vesicles as Model Cellular Membrane System**

Verónica Pérez-Luna,^1^ Carlos Moreno-Aguilar,^1^ José Luis Arauz-Lara,^1^ Said Aranda-Espinoza^1, *^ and Mildred Quintana^1,2, *^

1. Instituto de Física, Universidad Autónoma de San Luis Potosí, Manuel Nava 6, Zona Universitaria 78290, San Luis Potosí, SLP, México.
2. Centro de Investigación en Ciencias de la Salud y Biomedicina, Universidad Autónoma de San Luis Potosí, Av. Sierra Leona 550, 78210, San Luis Potosí, SLP, México.

*E-mail: [aranda@ifisica.uaslp.mx](mailto:aranda@ifisica.uaslp.mx) and [mildred@ifisica.uaslp.mx](mailto:mildred@ifisica.uaslp.mx)

**SUPPORTING INFORMATION**

All solvents and chemicals were purchased from Sigma-Aldrich and used without further purification. Lipids were purchased from Avanti Lipids. L-α-phosphatidylcholine (EggPC, #840051) and fluorescent dye L-α-phosphatidylethanol-amine N-(lissamine rhodamine B sulfonyl) (Egg-Rh PE, #810146) were used from stock solutions of 4 mg/ml in chloroform. Thin-MWCNTS were purchased from NANOCYL NC7000 ([www.nanocyl.com](http://www.nanocyl.com)).

The chemical composition of the samples was studied with X-ray Photoelectron Spectroscopy (XPS) using a Versaprobe PHI 5000 spectrometer from Physical Electronics, equipped with a monochromatic Al K α X-ray source with a 0.7 eV energy resolution.

In Figure 1, XPS spectra for the three MWCNTs samples are shown. Full core level spectra were collected, and evidence for C 1s peaks was found.

In the p-MWCNTs sample, shown in Fig.1b, the C 1s spectrum presents a central peak at a binding energy of 284.54 eV, which is assigned to photoelectrons emitted from sp^2^ carbon atoms (C=C and C-C bonds). This peak is in all MWCNTs. Secondary peaks are shown in Fig.1a for the ox-MWCNTs, which is generated by photoelectrons emitted from carbon atoms present in oxidizing groups: carboxyl (288.6 eV) and hydroxyl (285.92 eV). The chemical composition of p-MWCNTs is C (99.4 %) and O (0.6 %). For ox-MWCNTs, the chemical compositions are C (90.9 %) and O (9.1 %), while alk-MWNCTs contains C (97.5%) and O (2.5%).

Some TEM images were added in Fig. 1d-j to corroborate that the diameters and lengths of the MWCNTs were not altered due to their chemical functionalization; all samples maintained a diameter of approximately 9.5 nm and lengths of 1.5 µm.

**Figure 1.** Photoemission spectra and fit of the C 1S core level for all MWCNTs samples: a) ox-MWCNTs, b) p-MWCNTs, c) alk-MWCNTs. TEM micrographs of ox-MWCNTs (d and h), p-MWCNTs (f and i) and alk-MWCNTs (g and j).


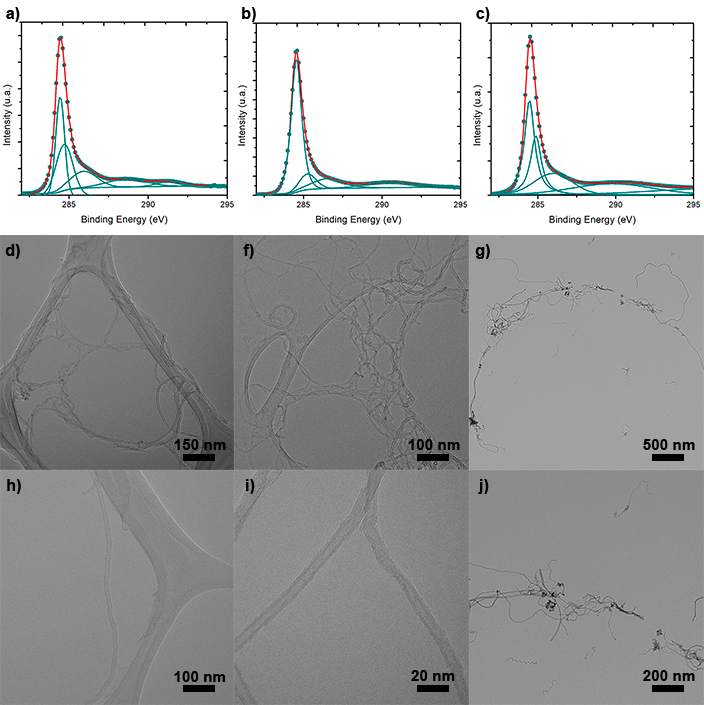


In the p-MWCNTs@GUVs systems, the quenching of the green fluorescence was observed during the confocal microscope scanning process, as shown in Fig. 2. The scanning process for each frame took about 15 seconds to complete, and the photobleaching of the FITC was noticed after one and a half minute of the beginning of the experiment.

**Figure 2.** Scanning confocal images of p-MWCNTs@GUVs systems during the time.


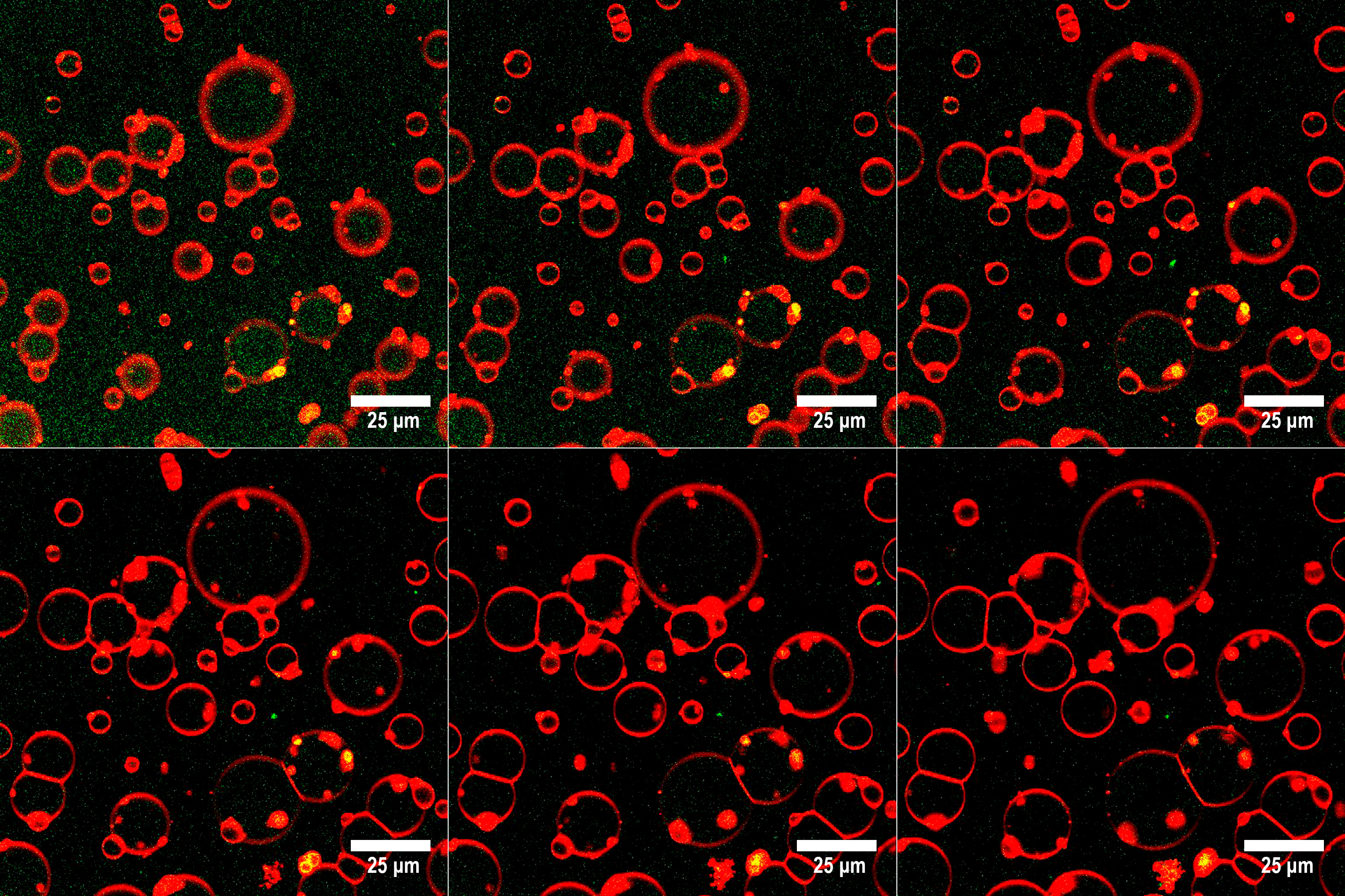


Videos of alk-MWCNTs@GUVs at a concentration of 2 µm/mL were added to show the diffusion of alk-MWCNTs through the GUVs membrane. All of the videos were recorded at 4fps using an Olympus CKX41 inverted phase contrast microscope with a Hamamatsu EM-CCD Digital Camera ImagEM-1K with a 40x objective.
